# Nonconcordance between Clinical and Head CT Findings: The Specter of Overdiagnosis

**DOI:** 10.1155/2013/314948

**Published:** 2013-10-01

**Authors:** Kelli N. O'Laughlin, Jerome R. Hoffman, Steven Go, Gelareh Z. Gabayan, Erum Iqbal, Guy Merchant, Roberto A. Lopez-Freeman, Michael I. Zucker, William R. Mower

**Affiliations:** ^1^Department of Emergency Medicine, Brigham & Women's Hospital, 75 Francis Street, Boston, MA 02115, USA; ^2^Harvard Medical School, 25 Shattuck Street, Boston, MA 02115, USA; ^3^University of California, Los Angeles (UCLA) David Geffen School of Medicine, 10833 Le Conte Avenue No. 12138, Los Angeles, CA 90095, USA; ^4^Department of Emergency Medicine, University of Missouri-Kansas City (UMKC) School of Medicine, 2411 Holmes Street, Kansas City, MO 64108, USA; ^5^Greater Los Angeles Veterans Affairs Healthcare System, 11301 Wilshire Blvd, Los Angeles, CA 90073, USA; ^6^Ronald Reagan UCLA, Emergency Medicine Center, 757 Westwood Plaza, Los Angeles, CA 90095, USA; ^7^Department of Medicine, University of California, Los Angeles (UCLA), 757 Westwood Plaza, Los Angeles, CA 90095, Irvine, USA; ^8^School of Medicine, University of California, Irvine, 1001 Health Sciences Road, 252 Irvine Hall, Irvine, CA 92697-3950, USA; ^9^UCLA Medical Center, 757 Westwood Plaza, Los Angeles, CA 90095, USA; ^10^Department of Emergency Medicine, University of Cincinnati, 234 Goodman Street, Cincinnati, OH 45219, USA; ^11^Emergency Medicine Center, University of California, Los Angeles (UCLA), 924 Westwood Blvd., Suite 300, Los Angeles, CA 90095, USA

## Abstract

*Background*. It is unclear whether history and physical examination findings can predict abnormalities on head computed tomography (CT) believed to indicate increased risk of lumbar-puncture- (LP-) induced brain herniation. The objectives of this study were to (1) identify head CT findings felt to be associated with increased risk of brain herniation and (2) to assess the ability of history and physical examination to predict those findings. *Methods*. Using a modified Delphi survey technique, an expert panel defined CT abnormalities felt to predict increased risk of LP-induced brain herniation. Presence of such findings on CT was compared with history and physical examination (H&P) variables in 47 patients. *Results*. No H&P variable predicted “high-risk” CT; combining H&P variables to improve sensitivity led to extremely low specificity and still failed to identify *all* patients with high-risk CT. *Conclusions*. “High-risk” CT is not uncommon in patients with clinical characteristics known to predict an absence of actual risk from LP, and thus it may not be *clinically relevant*. “Overdiagnosis” will be increasingly problematic as technological advances identify increasingly subtle deviations from “normal.”

## 1. Introduction

There is substantial evidence in the medical literature that lumbar puncture (LP) is extremely safe [1–3], particularly in the setting of patients who are immunologically normal and who do not manifest abnormal mental status, papilledema, or focal neurological deficit [3–5]. Nevertheless, many clinicians are concerned that LP could lead to tonsillar herniation [6–11] in patients with increased intracranial pressure (ICP), by decreasing cerebrospinal fluid pressure in the subarachnoid space, and thus creating a pressure gradient that could displace cerebral and brainstem structures [7, 12].

There are only a small number of reports, however, suggesting catastrophic deterioration related to LP [9, 13, 14], and even the validity of these observations has been questioned [15–17]. Nevertheless, despite the substantial evidence that few, if any patients, are at risk from LP, many clinicians worry about potential harm, and it has been suggested that head computed tomography (CT) be obtained prior to LP, in the hope that it might be able to identify the few patients in whom the procedure would purportedly be dangerous [5, 18]. There are no studies, however, which define what if any findings on head CT actually do predict increased risk of tonsillar herniation following LP [3, 4, 19]. 

We designed this study to assess the prevalence of “high-risk” CT findings in patients who undergo head CT for any reason and in particular whether and how often such findings occur in patients whose clinical presentations make it extremely unlikely that they would be harmed by LP. Because of the absence of any gold standard for “high-risk” CT findings, we convened an expert panel and asked its member to define what CT findings indicate increased risk of tonsillar herniation following LP; we then used clinical data that had been prospectively recorded on a large cohort of patients for whom CT had been ordered to assess the relationship between selected history and physical examination (H&P) findings and the presence of those CT characteristics defined by the expert panel as conferring increased risk. 

## 2. Methods

### 2.1. Ethics Statement

This study was approved by the University of California, Los Angeles (UCLA) Institutional Review Committee. Informed consent was not required as the clinical data collected was deidentified and did not impact clinical care.

### 2.2. Study Design

We designed this study in two parts. First, given the absence of any prior criterion standard regarding which if any head CT findings predict increased hazard from performance of LP, we convened an expert panel to determine if it was possible for them to agree on any set of head CT characteristics that could be defined as predicting “high risk” of LP-induced brain hemiation (Part I). Second, we used prospectively collected data from the large NEXUS 2 cohort of emergency department patients for whom head CT had been ordered, for whatever reason, to evaluate how closely clinical findings correlate with the presence or absence of any of these “high-risk” CT findings (Part II). We did not make any assumptions regarding what would have happened to any of the *individual* patients in our study had an LP been performed on them. (We did assume, on the other hand, based on substantial evidence from the literature, that at most a very few patients in this cohort might have been harmed by LP, particularly among that subset of patients who had been prospectively found not to have papilledema, altered mentation, or focal neurological findings.)

*Part I: The Modified Delphi Technique to Define Predictive Head CT Abnormalities*. We employed a modified Delphi survey technique [20–23] to allow a group of content experts, who perform LP and/or interpret head CT regularly, to generate a list of head CT abnormalities that they believed to predict high risk of herniation with LP and thus contraindicate this procedure. Of the 16 physicians asked to participate, 13 agreed. The panel included academic physicians in emergency medicine (2), internal medicine (1), infectious disease (1), neurology (4), neurosurgery (2), and radiology (3) including one general radiologist, one neuroradiologist, and one emergency radiologist), from four academic medical centers in the Midwest and the West Coast of the United States. 

 A structured e-mail survey regarding potential LP contraindications was sent to the panelists, each of whom then indicated his or her level of agreement (0 = “strongly disagree,” and 5 = “strongly agree”) with the following statement: “This CT finding should contraindicate performance of an LP,” for a series of possible CT findings (see Appendix information at “Information and Survey Sent to the Delphi Panel”). Narrative explanations of the experts' responses were solicited, and panelists were asked to suggest any additional CT findings they felt should contraindicate LP. For subsequent rounds, survey participants were asked to read the anonymous narrative explanations submitted by all of the panelists and then to rerate each CT finding. While three survey rounds are generally considered adequate to achieve a valid consensus opinion [24], when an additional potential LP contraindication was first suggested in the third round, one final survey round was added. Panelists were blinded to the identities of other panel members, but they were allowed to use outside references and to discuss the survey with colleagues. In order to maximize sensitivity, we included in the final list of “contraindications” any finding assigned a level of 4 or 5 by one-third of the panelists. 

*Part II: Study Subjects. *We included all of the 1737 patients for whom a head CT scan had been ordered at an academic, urban, level I emergency department (ED) between April 2006 and February 2007, as part of a prior study in which the treating clinician prospectively recorded the presence or absence of specific H&P findings at the time the CT was ordered and before it was actually done [25]. The only exclusion criterion was a history of a prior neurosurgical intervention. A neuroradiologist interpreted all CT images. Two physician reviewers, blinded to H&P findings, then independently classified the written head CT radiology reports as reflecting that the patient either was at increased risk (if *any* of the expert panel's increased-risk findings were present (Table 1)) or was not at increased risk (if *none* of these findings were present). When both reviewers independently agreed that the written report was definitive regarding presence or absence of the increased-risk findings, they were classified as such. In any case where *either* of the physicians was uncertain on how to classify this, based solely on the written report, the actual CT images were reviewed by an expert radiologist, who then prospectively characterized each study with regard to the presence or absence of increased-risk findings. If the emergency radiologist was uncertain, a senior emergency physician conducted an additional review of the CT images. To be as conservative as possible in these few remaining, this physician labeled the CT scan as showing “increased risk” only if any one of the following additional findings was present: dilation, enlargement or compression of the 4th ventricle or the brainstem, hydrocephalus, effaced cerebral sulci, local mass effect, or evidence of edema. 

### 2.3. Primary Data Analysis

We calculated the sensitivity, specificity, and positive and negative predictive values for each of the individual H&P findings in predicting the presence of increased risk of herniation with LP on head CT (as defined by the expert panel). We also analyzed the test characteristics of the complete neurologic examination and all aspects of the H&P, assuming that the presence of any single abnormal finding predicts increased risk as determined by CT. 

## 3. Results

### 3.1. Part I: The Modified Delphi Results

The expert panel ultimately agreed on five findings on head CT that were felt to increase risk of LP sufficiently so as to contraindicate performance of this procedure (Table 1). There was complete agreement on inclusion of subtentorial or tonsillar herniation, strong agreement on obliteration of the fourth ventricle, and majority agreement on lateral shift of midline structures and loss of basilar cisterns; a sufficient minority (at least one-third of the panel) felt that the obliteration of the superior cerebellar cistern and the quadrigeminal plate cistern should be included. Three other findings (isolated dilation of the temporal horns of the lateral ventricles, intracranial abscess in an immunocompromised patient, and Chiari I malformation with a tethered cord) had at least one vote for inclusion, but they were ultimately excluded because they were not endorsed by an adequate number of panelists.

### 3.2. Part II: Study Subjects Results

Of the 1737 patients initially included, 445 had abnormal head CTs. Of these, 122 were excluded because of a previous neurosurgical intervention, leaving 323 patients for the final analysis. The average age of study subjects was 57.8 years (range, 1–99 years), and 58% were male. Other demographic characteristics are listed in Table 2. 

**Figure 1:**
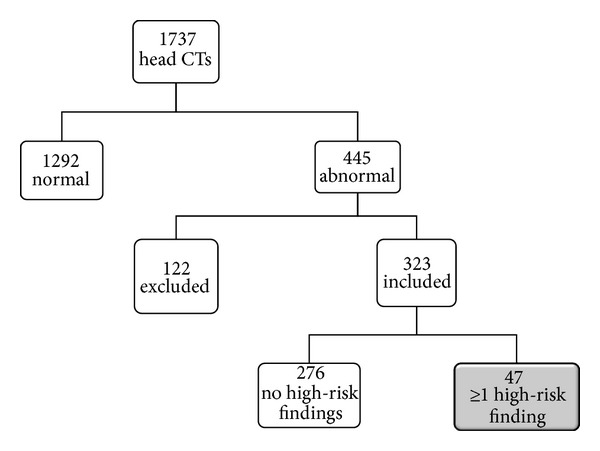
Study subject selection.

In categorizing the CT scan reports, both of the physician reviewers independently agreed that the written radiology report definitively indicated the presence, or absence, of the high-risk findings, in 68% of the cases. The emergency radiologist who then reviewed the actual CT images in the remaining 32% of cases was able to categorize findings as clearly present or absent in all but 2%; these few cases were then categorized by a senior emergency physician, using the conservative criteria described above (with cases classified as high risk only if they showed definitive dilation, enlargement or compression of the 4th ventricle or the brainstem, hydrocephalus, effaced cerebral sulci, local mass effect, or evidence of edema). Overall, 47 (14.6%) of the CT scans had at least one of the high-risk findings defined by the expert panel (Figure 1). 

None of the individual elements of H&P were sensitive in identifying patients with increased risk based on head CT findings (Table 3). The highest sensitivity for any single clinical characteristic was 68.9% (CI 53.4, 81.8), for presence of a focal neurological deficit on examination; this finding had a specificity of 73.5% (CI 67.8, 78.7). When the neurological examination was considered as a whole, with the presence of any single abnormality on examination compared with presence or absence of high-risk CT, sensitivity was still only 87.0% (CI 73.7, 95.1), and specificity was 39.3% (CI 33.5, 45.3). If the presence of any single abnormality on either history or physical examination was considered positive, sensitivity was increased to 95.7% (CI 85.5, 99.5), but specificity was further decreased to only 17.8% (CI 13.5, 22.9); this approach still failed to identify 2 of 47 patients with “high-risk” CT findings. 

## 4. Discussion 

There is substantial evidence in the medical literature that brain herniation secondary to lumbar puncture is extremely uncommon; this is particularly true among patients who do not have high-risk clinical findings, such as altered mentation or focal neurological deficits. Because of concerns about this catastrophic possibility, however, several authors have suggested that head CT scanning be performed prior to LP, to help identify patients at increased risk. 

Because of the absence of any prior criterion standard regarding “high-risk” CT findings, we performed Part I of our study to define a group of such findings about which experts would agree that they represent an increased risk of LP-induced brain herniation on head CT findings. Using standard accepted methodology of a modified Delphi technique, our expert panel was able to reach consensus, and at least one of their “high-risk” findings was present in about one of every seven patients in our cohort; a majority of these patients at “high risk” as indicated by their CT findings had clinical findings (as *prospectively* recorded) that suggested very little or no actual likelihood of danger from LP. The method used to characterize high-risk findings on CT scans (to rely on written radiology reports *only* when they were definitive and to have images prospectively reviewed when such written reports were not absolutely clear) was intentionally conservative and intended to bias against our hypothesis that CTs lead to overdiagnosis by showing “high-risk findings.” While we of course cannot know with any certainty what would or would not have happened to any individual in our cohort had he or she been subjected to LP, it is clear that this number is far larger than the rate of deterioration that should be expected amongst this group.

There are two very different possible explanations for this discordance between clinical findings in individual patients and “high-risk” elements on CT. One explanation is based on the *assumption* that when a technologically advanced test like CT identifies some problem not identified by clinical exam, the former always provides more accurate evidence of the patient's true condition; this type of reasoning is extremely widespread in modern medical practice. In the clinical scenario addressed by our study, that would mean that clinical findings are insufficient to identify patients in whom LP would be dangerous—and that CT should be performed routinely prior to LP—to identify a substantial group of patients with a clinically occult risk from this procedure. However, it is also possible that CT findings (as defined by this expert panel and as reflected in opinion papers in the medical literature [14]) are overly sensitive and identify a fairly large group of patients the large majority of whom are *not* in fact at any risk. It is not possible to say with certainty which of these interpretations is correct, based on this or any other currently available study. 

We believe that this disjunction between sophisticated technology and traditional diagnostic evaluation is not unique to head CT scanning and the risk of herniation and that the importance of our findings extends far beyond the narrow question of whether routine imaging (which also carries well-described medical and economic costs [3, 26, 27]), prior to LP, would on balance be beneficial or harmful. Modern technological approach to pulmonary embolism (PE), for example, has led to a vast increase in the number of patients given this diagnosis, but a concomitant vast decrease in the case-fatality rate associated with it [28], which appears to be largely independent of any benefit from advances in treatment. In addition, a recent decision analysis suggests that while an effort to identify PE in a cohort of reasonably low-risk patients using CT angiography may benefit a few patients, it will harm many more, increasing not only morbidity but also mortality [28]. Our observations about the use of CT to predict risk of brain herniation should thus be viewed in terms of this much larger question about technology in general and suggest that this question needs urgent attention from both the research community and the practicing physicians, since simply *assuming* that the former must be a better “gold standard” may in fact lead to major harm to patients [28, 29].

Although it is tempting to assume that technology is both more reliable and more accurate than clinical examination, there are several reasons to question this belief. First, there are numerous examples where some finding identified by some type of advanced technology is given the same name as a previously well-known clinical event, but it actually implies a far less dangerous clinical entity. As in the case of PE, described above, a hyphema diagnosed by slit-lamp examination is less worrisome (and should be approached differently) than a hyphema visible to the naked eye; similarly, CT-defined pneumothorax is clinically different than pneumothorax seen on a chest X-ray, acute myocardial infarction (MI) defined on the basis of troponin leak is not the same as the clinically apparent MI, and asymptomatic microscopic prostate “cancer” diagnosed by biopsy, after screening, implies a very different prognosis (and should be approached differently) than does cancer searched for and found because of clinical symptoms. 

With regard to the specific clinical issue raised in our study, a recent publication reported head CT “evidence of herniation” in many patients who were clinically entirely stable [30]. The meaning of such “herniation” is obviously different than is tonsillar herniation associated with catastrophic clinical deterioration. Because clinicians have long used clinical findings to decide who can safely undergo LP without a head CT and because with this approach tonsillar herniation rarely if ever occurs (especially in patients with normal mental status and no focal deficit), we believe that the CT criteria defined by our experts are likely to be overly sensitive and identify “risk” in many patients who could undergo LP with almost no chance of clinical deterioration. 

Since clinical brain herniation almost inevitably results in death, it is appropriate that CT criteria for safe performance of LP should err on the side of high sensitivity. On the other hand, failure to recognize that “findings” on head CT may not have the meaning traditionally attributed to the same “abnormalities” (“lesions” and “diseases”) identified on the basis of clinical condition could lead to dramatic overdiagnosis [29], which could in turn result in substantial harm to patients. In our scenario, this could lead to an insistence on routine CT scanning prior to LP, despite the long history of safe performance of LP without CT; furthermore, findings on those CT scans would almost certainly then lead to avoidance of LP in many patients in whom this test would not only be enormously safe but could also provide important diagnostic information.

In a broader sense, our study raises generic concerns about the danger of *assuming* that as technology advances, it will always provide a better and better diagnostic gold standard. We can easily envision, for example, a time when the *N*th generation CT scanners, using electron microscope-type resolution, might identify a “thyroid nodule” in just about everyone—which might be proved on biopsy to be “cancer” in many—even though the vast majority would never know about this “cancer” had the “advanced imaging” not been performed. The same could be true for “renal cysts”, or lesions on supermammography, or even “pulmonary emboli” in many if not all normal pulmonary arteries leading to obvious and profound conundrums about both the meaning of such findings and the appropriate way to separate disease, which might benefit from treatment, from overdiagnosis of normal (or at least clinically unimportant) variation. Surveillance bias has been described as “the more you look, the more you find,” [31]. The type of overdiagnosis we believe we have identified appears to be a closely related variant, where “the closer you look (with more and more powerful tools), the more you find.”

The head CT criteria defined by our panel as representing “increased risk” of herniation are based on expert opinion, rather than experimental evidence, which does not exist. Although many specialties were represented in the expert panel, not all regularly perform LP, which may have resulted in an overly conservative list of “contraindications.” Our expert panel also suffered a degree of expert attrition, with one neurosurgeon, one neurologist, and one neuroradiologist failing to complete the entire sequence of surveys. Peer pressure can influence expert panels [32], so this could conceivably have biased our results. 

Nevertheless, our study provides strong evidence that no H&P findings, alone or in combination, are adequately sensitive to detect head CT abnormalities believed by a panel of experts to predict enhanced potential for brain herniation during LP. Since clinical brain herniation is extremely rare following LP and these CT findings are far more common, it is likely that these criteria are overly sensitive and that their application to patients needs to be reconsidered. Furthermore, our study suggests that there is an urgent need to question the assumption that “advanced” technology defines the criterion standard when there is a clear disjunction between abnormalities defined clinically and “abnormalities” given the same name, despite an absence of clinical correlates, when identified by such technology.

## Figures and Tables

**Table 1:** Head CT abnormalities identified as contraindications to LP by the Delphi panel.

Head CT abnormalities	Percentage of the Delphi panel who rated the abnormality as 4 or 5 (out of 5)
Lateral shift of midline structures	73
Loss of basilar cisterns	73
Obliteration of the fourth ventricle	91
Subtentorial herniation or tonsillar herniation	100
Obliteration of the superior cerebellar cistern and the quadrigeminal plate cistern with sparing of the ambient cisterns	36

**Table 2:** Demographic and clinical characteristics of study subjects, *N* = 323.

Sex	
Male	188 (58.2)
Age	
Range	1 to 99
Mean	57.8
Median	62
Interquartile range (IQR)	47–87
Pediatric ages	
≤3	8
3 to 6	3
6 to 18	11
Race	
White	189
Black	17
Hispanic	57
Asian	22
Middle Eastern	17
Others/unknown	21

**Table 3:** Test characteristics of history and physical examination findings in relation to clinically abnormal head CTs.

	*N*	Sensitivity	CI	Specificity	CI	NPV	CI	PPV	CI
Blunt head injury	139/307	47.7	(32.5, 63.3)	55.1	(48.9, 61.3)	86.3	(80.2, 91.1)	15.1	(9.6, 22.5)
Dangerous mechanism	105/285	33.3	(19.6, 49.6)	62.6	(56.1, 68.7)	84.4	(78.3, 89.4)	13.3	(7.5, 21.4)
Vomiting	23/318	8.9	(2.5, 21.2)	93	(89.4, 95.8)	86.1	(81.6, 89.3)	17.4	(5.0, 38.7)
Coagulopathy	36/280	8.3	(1.8, 22.5)	86.5	(81.5, 90.5)	86.5	(81.5, 90.5)	8.3	(1.8, 22.5)
Skull fracture	21/319	11.6	(3.8, 24.6)	94.2	(90.7, 96.64)	86.9	(82.6, 90.5)	23.8	(8.2, 47.2)
Scalp hematoma	81/319	26	(14.2, 41.1)	74.7	(69.1, 79.8)	85.7	(80.6, 89.9)	14.8	(7.9, 24.5)
Neuro deficit	102/313	68.9	(53.4, 81.8)	73.5	(67.8, 78.7)	93.4	(89.1, 96.3)	30.4	(21.7, 40.3)
ALOC	134/316	67.4	(52.0, 80.5)	61.9	(55.8, 67.7)	91.8	(86.8, 95.3)	23.1	(16.3, 31.2)
Abnormal behavior	100/314	53.3	(37.9, 68.3)	71.8	(66.0, 77.1)	90.2	(85.4, 93.8)	24	(16.0, 33.6)
No spontaneous eye opening	61/316	34.1	(20.5, 49.9)	83.1	(78.1, 87.3)	88.6	(88.1, 92.3)	24.6	(14.5, 37.3)
Not oriented	189/305	65.1	(49.1, 79.0)	66.4	(60.3, 72.1)	92.1	(87.3, 95.5)	24.1	(16.7, 33.0)
Not following commands	76/310	43.2	(28.34, 59.0)	78.6	(73.2, 83.4)	89.3	(84.6, 93.0)	25	(15.8, 36.3)
Amnestic	28/224	18.5	(6.3, 38.1)	88.3	(83.0, 92.5)	88.8	(83.5, 92.8)	17.9	(6.1, 36.9)
Combined criteria	271/322	95.7	(85.5, 99.5)	17.8	(13.5, 22.9)	96.1	(86.5, 99.5)	16.6	(12.4, 21.6)
Abnormal neuro exam	207/321	87	(73.7, 95.1)	39.3	(33.5, 45.3)	94.7	(88.9, 98.0)	19.3	(14.2, 25.4)
Abnl neuro exam or vomiting	217/321	87	(73.7, 95.1)	36	(30.3, 42.0)	94.3	(88.0, 97.9)	18.5	(13.6, 24.4)

*N*: number, CI: confidence interval, PPV: positive predictive value, NPV: negative predictive value, Abnl: abnormal, and Neuro: neurological.

## Appendix

### Information and Survey Sent to the Delphi Panel

Dear Colleague:

Thank you for agreeing to be part of an expert panel to help determine CT contraindications to LP. This is round 1 of 3 questionnaires regarding the topic.

For the following 3 items:

1. *Please indicate your level of agreement that the CT finding listed is a contraindication for performing an lumbar puncture* using a 5 point scale (0 indicates strong disagreement and 5 strong agreement).
2. Please *explain the reasons for your choice* in the space provided.
3. Please *add any additional CT findings* that are contraindications to LP you feel should be on the list.

*Please return this questionnaire by email or fax*.

Your Name: ————————————————

1. The CT finding of a lateral shift of the midline structures is a contraindication to performing an LP.
  Strongly disagree 0 1 2 3 4 5 Strongly agree
  Your level of agreement with statement #1: —————
  Please explain the reason(s) for your answer:
2. The CT finding of a loss of the basilar cisterns is a contraindication to performing an LP.
  Strongly disagree 0 1 2 3 4 5 Strongly agree
  Your level of agreement with statement #2: —————
  Please explain the reason(s) for your answer:
3. The CT finding of an obliteration of the fourth ventricle is a contraindication to performing an LP.
  Strongly disagree 0 1 2 3 4 5 Strongly agree
  Your level of agreement with statement #3: —————
  Please explain the reason(s) for your answer:
4. The CT finding of an obliteration of the superior cerebellar cistern and the quadrigeminal plate cistern with sparing of the ambient cisterns is a contraindication to performing an LP.
  Strongly disagree 0 1 2 3 4 5 Strongly agree
  Your level of agreement with statement #4: —————
  Please explain the reason(s) for your answer:
5. Please suggest any additional CT findings that are contraindications to LP that you feel should be included in this list, and briefly explain why.
